# Two Cases Diagnosticated as Milk Fever

**Published:** 1882-01

**Authors:** B. C. Seers


### V.—TWO CASES DIAGNOSTICATED AS MILK FEVER.

REPORTED BY B. C. SEERS.

*Milk Fever.*—This disease is probably as fatal as any which the dairyman has to contend with. Our habit of feeding heavily on Indian corn and other rich food, for eight months in the year, makes our cows more liable to this malady. The symptoms of this affection are generally an entire stoppage of the flow of milk, accompanied by all the symptoms of fever, which varies in intensity. After a few hours the animal acquires a staggering gait, with a want of muscular power in the posterior extremities.

I have found in the majority of cases no remedy so serviceable, provided the case is seen in an earlier stage, as free bleeding, say from five to ten quarts, according to the size and flesh of the animal, and the type of fever. The amount of diminution in the milk secretion should also be considered in reference to treatment.

We have had two similar cases during the past summer, which we treated alike. The result, however, in the two cases was not alike; one case recovered, and the other died.

I report the two cases, and subject the same to criticism, on account of the trouble which we have in dealing with the severe cases.

CASE I.—The first animal attacked was a large common cow, who calved rather early, and at the time of her illness was secreting less than the average quantity of milk. Without apparent cause, some two or three weeks after calving, the flow of milk suddenly stopped; the horns and ears were quite warm at the base, but unusually cold at the tips. The mouth was very hot, the eyes red, the Schneiderian membrane dry, and the nostrils were partially filled with a scaly discharge. The cow staggered about, and soon fell down from loss of power in the posterior extremities.

At noon, bled the cow, allowing some six quarts of blood to escape, and gave, per orem, one ounce of the nitrate of potass (saltpetre). At four o'clock P. M. of the same day, the cow still continued to be very sick; the breathing was hurried and short. She noticed but very little around, but drank a little water. At dark, no better. Nine o'cloek P. M., the animal noticed my approach, and was better. The next morning she was able to walk about, began to graze, and in two or three days was giving as much milk as ever, and has remained well up to date.

Case II.—This animal was a grade Jersey, about eight years old, in fair flesh; she was a good milker, giving a fair quantity of rich milk. This cow was taken sick out in the field two or three days after calving. The following notes were taken at the time. Saturday morning, September 10th, 6 o'clock A.M., I found the cow down and unable to rise. I bled the animal, allowing three or four quarts of blood to escape. The hemorrhage was arrested when a change of color was noticed in the blood from a dark to a light red. To this animal one ounce of the nitrate of potassium (saltpetre) was given, but in the crystaline form. At 2 o'clock P.M., the cow was worse, pulse 80 per minute. Bled again, allowing four or five more quarts of blood to escape. At this point the pulse softened very suddenly, and the hemorrhage ceased spontaneously. The animal now appeared very sick, the conjunctiva no longer sensitive to the touch, and the head laying to one side as if the cow was dead. Administered one pint spiritus mallati (apple whiskey), and one ounce pulv. zinzebar (ginger). This dose was repeated at 7 o'clock P.M. At this time the animal could scarcely swallow; pulse, 80 per minute, regular but very feeble. The secretion of milk was not totally arrested, but some flowed during the whole sickness.

Sunday morning, appeared a little better, noticed attendants and swallowed readily. Gave half pint spiritus mallati, one ounce of pulv. zinzebar, and one half quart of raw oleum lini (linseed oil). Drank some water during the day. P.M., still better, gave another pint of whiskey and had her covered with a blanket.

Monday morning, cow walked a half mile to the home barn; bowls remained constipated. Gave another quart of oil, which moved the bowels freely. Fever still continues; no appetite; gave but little milk. Ordered bottle of old hard cider, with one drachm of pulv. zinzebar, night and morning. Tuesday, ordered condition powders, such as are commonly given to horses, one to be given twice daily. She, however, never ruminated or ate anything. The fæcal discharges continued to be dark and thin. The fever continued and the animal died on the fifteenth day of the disease.

Now, why did not the treament which cured the first cow cure the second? Was the dose of saltpetre too heavy? Should the bleeding have been carried further at first (I think so), and did the cow die of typhoid fever? If so, what would have been the remedy?

Any or all of these questions I should be glad to have answered by yourself or correspondents, together with a full criticism of the treatment, or so much of it as you may think of enough general interest to print.

BLOOMING GROVE FARM, Orange Co., N. Y.

[The first case, as above reported, differs markedly from milk fever. It, however, simulates the condition known as congestion of the brain and spinal cord with the paralysis common to that condition.

The exciting cause of the first case was, in all probability, engorgement of the rumen with food and a slight catarrhal fever.

Milk fever is not usually developed three weeks after calving, neither is the recovery so rapid. Very few animals can get up even after the second day, much less can they walk.

The second case, as reported, was probably milk fever. Should judge the case was rather far advanced for such heroic measures as bleeding, etc. Generally such cases do better under the *action* of stimulants and tonics.

The difference in the result in the two cases was probably due to the original diseases being different. We should be inclined to regard the second case as true milk fever, and not typhoid, although it may have assumed a typhoid character.

In reference to the use of the nitrate of potass, we do not see the indication for its use. It is one of the most powerful and irritating saline diuretics, but in this case no reference is made to an interference with the functions of the kidney. Therefore, we should positively say that it was counter indicated. In case there was reason to use a diuretic, we would in all such cases advise the use of non-irritating diuretics in preference to the irritating. —ED.]

